# Conventional MR and DW imaging findings of cerebellar primary CNS lymphoma: comparison with high-grade glioma

**DOI:** 10.1038/s41598-020-67080-9

**Published:** 2020-06-19

**Authors:** Ye-Xin He, Chong-Xiao Qu, Yuan-Yan He, Jia Shao, Qiang Gao

**Affiliations:** 1 Department of Radiology, Shanxi Provincial People’s Hospital, Affiliated People’s Hospital of Shanxi Medical University, Taiyuan, 030012 China; 2 Department of Pathology, Shanxi Provincial People’s Hospital, Affiliated People’s Hospital of Shanxi Medical University, Taiyuan, 030012 China; 3 Department of Medical Imaging, Shanxi Medical University, Taiyuan, 030001 China

**Keywords:** Neurology, Oncology, Diagnosis, Medical imaging

## Abstract

Primary central nervous system lymphomas (PCNSLs) and high-grade gliomas (HGGs) arising in the cerebellum is extremely low, making the differential diagnosis difficult or even impossible. The purpose of this study was to define the MR features of cerebellar PCNSL in immunocompetent patients, and to determine whether a combination of conventional MR and DW imaging can assist in the differentiation of PCNSLs and HGGs. Twelve PCNSLs and 15 HGGs confirmed by pathological analysis were retrospectively identified. The apparent diffusion coefficient (ADC) and conventional MRI parameters were compared for differences between PCNSL and HGG groups using the independent sample t test or chi-square test. Both ADC_min_ and ADC_total_ values were lower in the PCNSL group than those in the HGG group (ADC_min_: 0.53 × 10^−3^ vs. 0.83 × 10^−3^ mm^2^/sec, P < 0.001; ADC_total_: 0.66 × 10^−3^ vs. 0.98 × 10^−3^ mm^2^/sec, P = 0.001). As for conventional MR features, there were significant difference in the tumor size, enhancement patterns, the presence of cystic changes, edema degree and streak-like edema (all P < 0.01); but there were no significant difference in lesion type, the presence of bleeding, and involvement of brain surface between two groups (P = 0.554, 0.657 and 0.157, respectively). The results revealed that several conventional MR features, including enhancement patterns, branch-like enhancement and streak-like edema may be useful for the differentiation of PCNSL and HGG in cerebellum and, when combined with ADC values, further improve the discriminating ability.

## Introduction

Primary central nervous system lymphomas (PCNSLs) are relatively rare, accounting for 1% of all intracranial tumors, and occurs in supra-tentorial location in majority of patients with posterior fossa as a location of tumor only in 7% of the cases^1^. Both PCNSLs and high grade gliomas (HGGs) are malignant tumors in adults and exceedingly rare in cerebellum^2^. Accurate preoperative diagnosis is often crucial because the management and prognosis of these tumors are substantially different. For example, patients with HGGs are almost always treated by surgical resection^3^, while patients with suspected PCNSLs are managed primarily with chemotherapy or radiation therapy after stereotactic biopsy^4,5^. Therefore, it is of great value to accurately distinguish the two before clinical treatment.

PCNSLs and HGGs arising in the cerebellum are extremely low, making the differential diagnosis difficult or even impossible. Although conventional magnetic resonance imaging (MRI) has shown considerable potential in the diagnosis and follow-up monitoring of brain tumors, the MRI features of PCNSLs and HGGs are changeable and overlapping. Due to their extreme rarity, it is sometimes difficult to differentiate them from other tumors in the cerebellum by conventional MRI. Therefore, awareness of the MR findings of these uncommon tumors in cerebellum is essential for prompt diagnosis.

Diffusion-weighted imaging (DWI) has been widely used in brain tumor evaluation, which is considered the most sensitive method for detecting differences in the molecular water diffusion of living tissues^6^. The apparent diffusion coefficient (ADC) value derived from DWI has been reported to be inversely correlated with cellularity in tumors^7–9^. Previous studies have shown that ADC values can help differentiate PCNSLs from HGGs^10–14^. The median minimum ADC (ADC_min_) values of PCNSLs were significantly lower than those of GBMs (0.73 × 10^−3^ vs. 0.89 × 10^−3^ mm^2^/sec, respectively, P < 0.0001)^13^. At a ADC_min_ cutoff value of 0.82 × 10^−3^ mm^2^/sec, it was possible to differentiate PCNSLs from GBMs with 73.0% sensitivity, 72.2% specificity, and 0.75 AUC^13^. However, whether a DWI analysis is effective in distinguishing the PCNSLs from HGGs in cerebellum remain largely unknown.

Here, we retrospectively analyze the conventional MR and DW imaging characteristics of cerebellar PCNSLs and HGGs from adult group, and determine whether a combination of conventional MR and DW imaging can assist in the differentiation of PCNSLs and HGGs.

## Materials and Methods

This retrospective study complies with the Declaration of Helsinki and research approval was granted from the Ethics Committee of Shanxi Provincial People’s Hospital, and informed consent was waived.

### Patient selection

Between May 2012 and December 2019, 27 patients who underwent a preoperative brain MRI and DWI for newly diagnosed PCNSL (n = 12) or HGG (n = 15) based on pathological evaluation were retrospectively reviewed. The inclusion criteria were as follows: pathologically confirmed PCNSL or HGG; no previous brain biopsy or surgery; available preoperative MRI data including sequences of T1, T2, FLAIR, T1 + C, and DWI; no steroid administration before MRI.

### Brain MRI protocol

The whole brain MRI examinations were performed on a 3.0-T MRI system (MAGNETON Trio, Siemens Healthcare Gmbh, Erlangen, Germany) with a 45-mT/m maximum gradient capability and an twelve-channel head coil (Siemens Medical Systems). Conventional MR and DW sequences of brain were performed in regular sequence during the same examination.

Conventional MRI sequences included fast low angle shot (FLASH) T1-weighted imaging in the transverse plane (TR/TE,440 ms/2.46 ms; matrix size, 256 × 256; field of view (FOV), 22 cm × 22 cm; Average, 1; slice thickness, 4.5 mm; gap, 0.45 mm), and sagittal planes (TR/TE, 360 ms/2.53 ms; matrix size, 256 × 256; FOV, 22 cm × 22 cm; Average, 1; slice thickness, 4 mm; gap, 0.4 mm), T2-weighted turbo spin echo in the transverse planes (TR/TE, 7,140 ms/98 ms; matrix size, 384 × 384; FOV, 22 cm × 22 cm; Average, 2; slice thickness, 4.5 mm; gap, 0.45 mm), and fat-saturated fluid-attenuated inversion recovery (FLAIR) in the transverse plane (TR/TE, 6,500 ms/91 ms; matrix size, 256 × 256; FOV, 22 cm × 22 cm; Average, 1; slice thickness, 4.5 mm; gap, 0.45 mm).

DWI sequence (b = 1000 sec/mm^2^) was performed using a single-shot diffusion-weighted spin-echo echo-planar sequence. In total, 19 axial slices covering the entire brain were obtained with the following parameters: FOV, 23 cm × 23 cm; slice thickness, 5 mm; slice gap, 1.5 mm; TR, 3,200 ms; TE, Minimum; and matrix 192 × 192. The total scan time was approximately 1 minute and 2 seconds.

Finally, a contrast-enhanced T1-weighted FLASH sequence was performed in the transverse, sagittal, and coronal planes with fat sat (TR/TE, 582 ms/2.46 ms; matrix size, 256 × 256; FOV, 22 cm × 22 cm; Average, 1; slice thickness, 4.5 mm; gap, 0.45 mm for coronal planes, other parameters for transverse and sagittal planes were the same as noncontract sequence), following a bolus injection of 0.1 mmol/kg of gadodiamide injection (Omniscan; GE Healthcare, Co.Cork, Ireland).

### MRI data processing and quantitative analysis

All data were analyzed and processed on Multi Modality Workplace (Siemens Healthcare Gmbh, Erlangen, Germany). Conventional MRI features and ADC values were analyzed or measured independently by two experienced radiologists, Y.-X.H. and C.X., with 15 and 20 years of experience in Neurological MRI, respectively. The observers were told before the review that the patients had cerebellar tumors, but they were blinded to the definite pathological results.

The conventional MR images were analyzed to determine the tumor size, lesion type, contrast enhancement, cystic change, bleeding, edema, and brain surface involvement. The maximum diameter of the tumor was measured at the level where the tumor appeared largest on the cross-sectional image. The mean diameter was calculated by (a + b + c)/3, and the maximal cross-section was used to measure the long (a) and short (b) diameters, longest diameter of tumor measured as c in sagittal or coronal plane. Lesion types were classified based on tumor number and boundary, and recorded as solitary demarcated lesion, multiple demarcated lesions, solitary infiltrative type and diffuse infiltrative lesions^2^. Contrast enhancement was homogeneous or heterogeneous, and homogeneous was further subclassified as branch-like enhancement, appeared as branch-like abnormal enhancement in the periphery of tumor, representing white matter fiber infiltration. The cystic change of the tumors was considered positive if intratumor areas of high T2 signal were non-enhancing on enhanced T1 images. Tumor bleeding is defined as the presence of high T1 signal within tumor area. Edema was scored as mild (extending less than half diameter from the lesion margin), severe (extending more than one diameter from the lesion margin) or moderate (fall in between mild and severe), and further defined as streak-like edema based on the edema shape. The presence of brain surface involvement were recorded as no reaching brain surface, reaching brain surface(contact with a cerebrospinal fluid surface), meningeal infiltration or ependymal infiltration (outside any point of contact of a parenchymal lesion with the subarachnoid space). Decisions concerning the conventional MR findings were reached by consensus.

The ADC values was calculated by fitting the b_0_ image and DWI at each b value other than 0 sec/mm^2^ into the mono-exponential equation (Eq. (1))^15^, where S_b_ is the diffusion weighted signal intensity for the b-value, and S_0_ is the signal intensity obtained with the b_0_ value. ADC maps were calculated on a pixel-by-pixel basis.

1 $${{\rm{S}}}_{b}/{{\rm{S}}}_{0}=\exp (-{\rm{b}}\times {\rm{ADC}})$$

The minimum ADC (ADC_min_) and whole tumor ADC (ADC_total_) values were measured based on region of interests (ROIs) and volume of interest (VOI), respectively. First, the observer reviewed the conventional MR images carefully to determine the slice containing the relatively largest cross-sectional area of the tumor solid part. Next, DWI data were analyzed. For measuring the ADC_min_, two circular region of interests (ROIs) were manually drawn using an electronic cursor in the slice containing the largest cross-sectional area, which were placed to include the solid tumor elements by defining ROIs based on the relatively high signal intensity on the DW image (b = 1000 sec/mm^2^), or the relatively low ADC value in the ADC map, avoiding large vessels, hemorrhagic, cystic and necrotic areas. The mean ROI area was 0.07 cm^2^. For measuring the ADC_total_, a volume of interest (VOI) was drawn manually around the entire cross-sectional tumoral region in three consecutive slices containing the largest cross-sectional area on DW images in a slice by slice method. Care was taken to exclude adjacent tissues, attempting to maintain an approximate distance of 1–2 mm away from the tumor margin to minimize the partial volume phenomenon^16^. The mean ADCmin and ADC_total_ values within the ROIs or VOI were obtained, respectively.

### Statistical analysis

IBM SPSS 20.0 software (IBM Corp, Chicago, IL, USA) was used for statistical analysis. The Kolmogorov–Smirnov (K–S) test was used to assess the normality of data distributions. Numerical variables with normal distribution were denoted as mean and standard deviation. Between-group comparisons of conventional MRI findings (including lesion types, enhancement, edema characteristics, presence of cystic change, bleeding, and brain surface involvement) were conducted using the chi-square test or Fisher’s exact test. Differences in the ADC_min_ and ADC_total_ values between PCNSL and HGG patients were evaluated using independent sample *t*-tests. Intra-class correlation (ICC) was used to estimate the agreement for ADC values from two readers. The ICC was interpreted as poor if it was less than 0.4, moderate when it was ≥0.4 but <0.75, and good when it was >0.75. *P* < 0.05 indicated a statistically significant difference.

## Results

### Demographic data

Table 1 exhibits the demographic characteristics of the patients. The final study population was comprised of 27 patients with newly diagnosed patients according to the pathological results including 12 PNCSLs (5 men, 7 women; mean age, 54.3 ± 9.4 years) and 15 HGGs (7 men, 8 women; mean age, 49.9 ± 22.1 years). The major clinical manifestations of the patients include headache and/or nausea (63.0%; 17 of 27 patients), dizziness (18.5%; 5 of 27), cranial nerve dysfunction (11.1%; 3 of 27) and paresis in 2 patients (10.0%).

**Table 1:** Clinical and demographic characteristics of cerebella PCNSL and HGG patients.

Patient characteristics	PCNSL (n = 12)	HGG (n = 15)
**Age** (mean ± sd, yrs)	54.3 ± 9.4	49.9 ± 22.1
**Sex** (M:F)	5:7	7:8
**Initial symptoms** (n)		
Headache/ nausea	7	10
Dizziness	3	2
Cranial nerve dysfunction	1	2
Paresis	1	1
**Pathologic procedure** (n)		
Biopsy	2	0
Resection	10	15
**Pathological types** (n)	diffuse large B cell lymphoma (11)	glioblastoma (9)
	extranodal NK/T cell lymphoma, nasal type (1)	anaplastic astrocytoma (5)
		anaplastic oligodendroglioma (1)

**Note**: **PCNSL** = primary central nervous system lymphoma; **HGG** = high grade glioma.

According to the pathological analysis, 11 patients had diffuse large B cell lymphoma, 1 extranodal NK/T cell lymphoma and 15 HGGs (glioblastoma (n = 9), anaplastic astrocytoma (n = 5) and anaplastic oligodendroglioma (n = 1)) (Table 1).

### Comparison of ADC values in PCNSL and HGG patients

**Figure 1:**
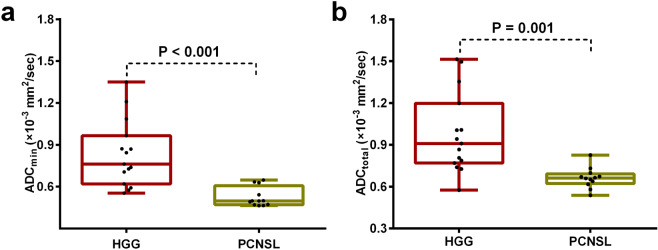
Box plots for ADC_min_ **(a**) and ADC_total_ **(b**) values in cerebellar high grade gliomas (HGG) and primary central nervous system lymphoma (PCNSL). HGG show significantly higher ADC_min_ and ADC_total_ values than do PCNSL (P < 0.05). **ADC**_**min**_ = minimum apparent diffusion coefficients, **ADC**_**total**_ = apparent diffusion coefficients of total tumor.

Comparisons of ADC values between PCNSL and HGG groups were shown in Table 2 and Fig. 1. Both ADC_min_ and ADC_total_ values were lower in the PCNSL group than those in the HGG group from two readers (reader 1, ADC_min_: 0.53 × 10^−3^ vs. 0.83 × 10^−3^ mm^2^/sec, P < 0.001; ADC_total_: 0.66 × 10^−3^ vs. 0.98 × 10^−3^ mm^2^/sec, P = 0.001; and reader 2, ADC_min_: 0.54 × 10^−3^ vs. 0.87 × 10^−3^ mm^2^/sec, P < 0.001; ADC_total_: 0.66 × 10^−3^ vs. 1.03 × 10^−3^ mm^2^/sec, P < 0.001).

**Table 2:** Comparison of ADC value and conventional MR findings for cerebella PCNSL and HGG.

Variable	PCNSL (n = 12)	HGG (n = 15)	*P*-value
**ADC** (mean ± sd, ×10^−3^ mm^2^/sec)			
**Reader 1**			
ADC _min_	0.53 ± 0.07	0.83 ± 0.24	<0.001^*^
ADC_total_	0.66 ± 0.07	0.98 ± 0.29	0.001^*^
**Reader 2**			
ADC _min_	0.54 ± 0.07	0.87 ± 0.28	<0.001^*^
ADC_total_	0.66 ± 0.07	1.03 ± 0.25	<0.001^*^
**Size** (mean ± sd, cm)			
Maximum diameter	2.61 ± 1.00	3.85 ± 0.54	0.001^*^
Mean diameter	2.22 ± 0.82	3.22 ± 0.41	0.002^*^
**Lesion type**			0.554
Solitary demarcated	2 (16.7)	5 (33.3)	
Multiple demarcated	2 (16.7)	0 (0)	
Solitary infiltrative	7 (58.3)	6 (40.0)	
Diffuse infiltrative	1 (8.3)	4 (26.7)	
**Contrast enhancement** - n (%)			<0.001^*^
Homogeneous	12 (100.0)	0 (0)	
Heterogeneous	0 (0)	15 (100.0)	
Branch-like enhancement	8 (66.7)	0 (0)	
**Cystic change** - n (%)			<0.001^*^
Yes	2 (16.7)	14 (93.3)	
No	10 (83.3)	1 (6.7)	
**Signs of bleeding** - n (%)			0.657
Yes	4 (33.3)	5 (33.3)	
No	8 (66.7)	10 (66.7)	
**Edema characteristics** - n (%)			0.003
Mild	3 (25.0)	13 (86.7)	
Moderate	6 (50.0)	1 (6.7)	
Severe	3 (25.0)	1 (6.7)	
**Streak-like edema**	7 (58.3)	0 (0)	0.001^*^
**Involvement of brain surface** - n (%)			0.157
No reaching brain surface	4 (33.3)	1 (6.7)	
Reaching brain surface	6 (50.0)	10 (66.7)	
Meningeal infiltration	1 (8.3)	2 (13.3)	
Ependymal infiltration	1 (8.3)	2 (13.3)	

^*^P < 0.05 indicated a statistically significant difference between groups; **PCNSL** = Primary central nervous system lymphoma; **HGG** = high grade glioma.

### Comparison of conventional MR findings in PCNSL and HGG patients

**Figure 2:**
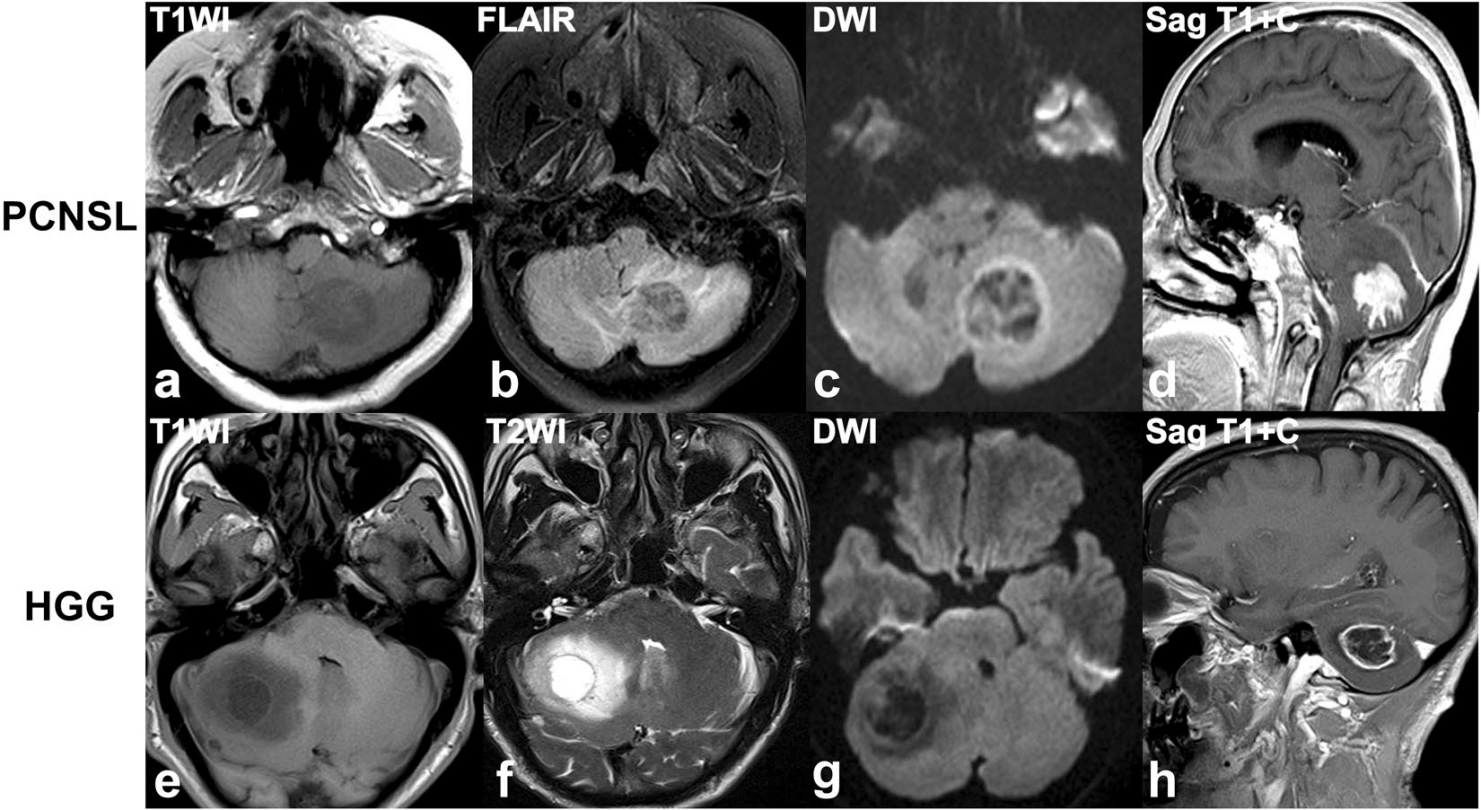
(**a–d**) A 58-year-old woman with a diffuse large B cell lymphoma in left cerebellum, presenting with intermittent dizziness, nausea and vomiting. **(e–h)**. A 70-year-old man with a anaplastic astrocytoma in right cerebellum, presenting with headache, dizziness, nausea and vomiting. Axial T1W (**a,e**), axial fat-saturated FLAIR (**b**) and T2W (**f**), diffusion-weighted (b = 1000 sec/mm^2^) (**c,g**), and sagittal contrast-enhanced T1W (**d,h**) images reveal a homogeneously enhancing tumor in the left cerebellum (**a–d)**, with moderate streak-like edema (**b**), peripheral diffusion restriction (**c**), and branch-like enhancement (**d**, arrow), and a heterogeneously enhancing tumor in the right cerebellum (**e–h)**, with severe edema (**f**), hypo- in centre and iso-intensity diffusion signal in periphery of tumor (**g**), central cystic change and peripheral ring-like enhancement (**h**).

**Figure 3:**
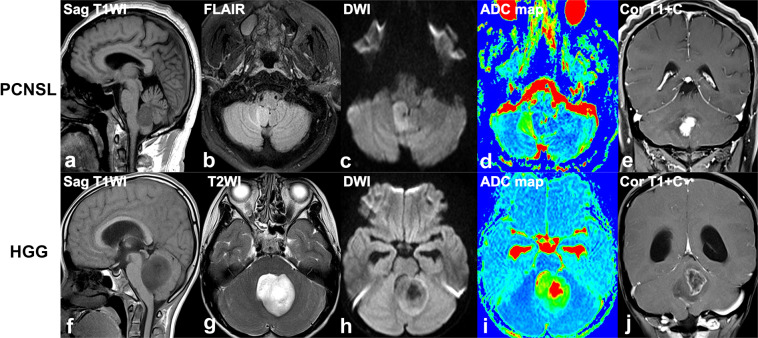
(**a–e**). A 52-year-old man with a diffuse large B cell lymphoma in right cerebellum, presenting with intermittent dizziness. **(f–j)** A 11-year-old girl with a anaplastic astrocytoma in vermis region of cerebellum, presenting with headache, dizziness, nausea and vomiting for two weeks. Sagittal T1W (**a,f**), axial fat-saturated FLAIR (**b**) and T2W (**g**), diffusion-weighted (b = 1000 sec/mm^2^) (**c,h**), apparent diffusion coefficient (ADC) map (**d,i**), coronal contrast-enhanced T1W (**e,j**) images reveal a homogeneously enhancing tumor in the right cerebellar vermis region (**a–e)**, with mild streak-like edema (**b**), diffusion restriction with low ADC value (**c,d**), and branch-like enhancement (**e**), and a heterogeneously enhancing tumor in cerebellar vermis region (**e-j)**, with mild edema (**f**), hypo- in centre and iso-intensity diffusion signal in periphery of tumor with relatively high ADC value **(h,i**), eccentric cystic change and peripheral ring-like enhancement (**h**).

**Figure 4:**
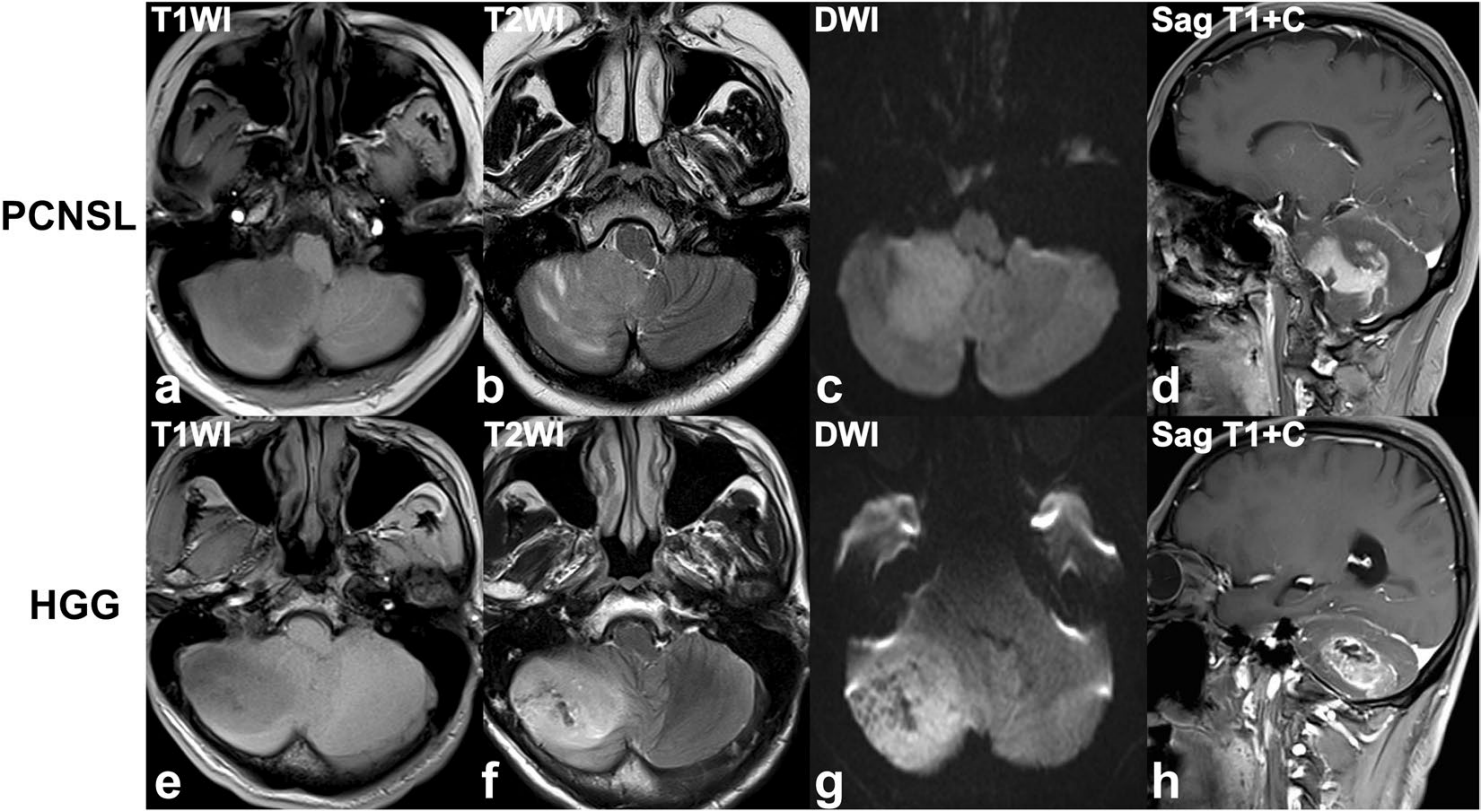
(**a–d**). A 35-year-old woman with a diffuse large B cell lymphoma in the right cerebellum, presenting with intermittent nausea and vomiting, unclear vision in right eye. **(e–h)** A 56-year-old man with a glioblastoma in right cerebellum, presenting with headache, dizziness, nausea and vomiting. Axial T1W (**a,e**), T2W (**b,f**), diffusion-weighted (b = 1000 sec/mm^2^) (**c,g**), and sagittal contrast-enhanced T1W (**d,h**) images reveal a heterogeneously enhancing tumor in the right cerebellum (**a–d)**, with moderate streak-like edema (**b**), diffusion restriction (**c**), and branch-like enhancement (**d**), and a heterogeneously enhancing tumor in the right cerebellum (**e–h)**, with mild edema (**f**), diffusion restriction (**g**), eccentric cystic change and heterogeneous enhancement (**h**).

**Figure 5:**
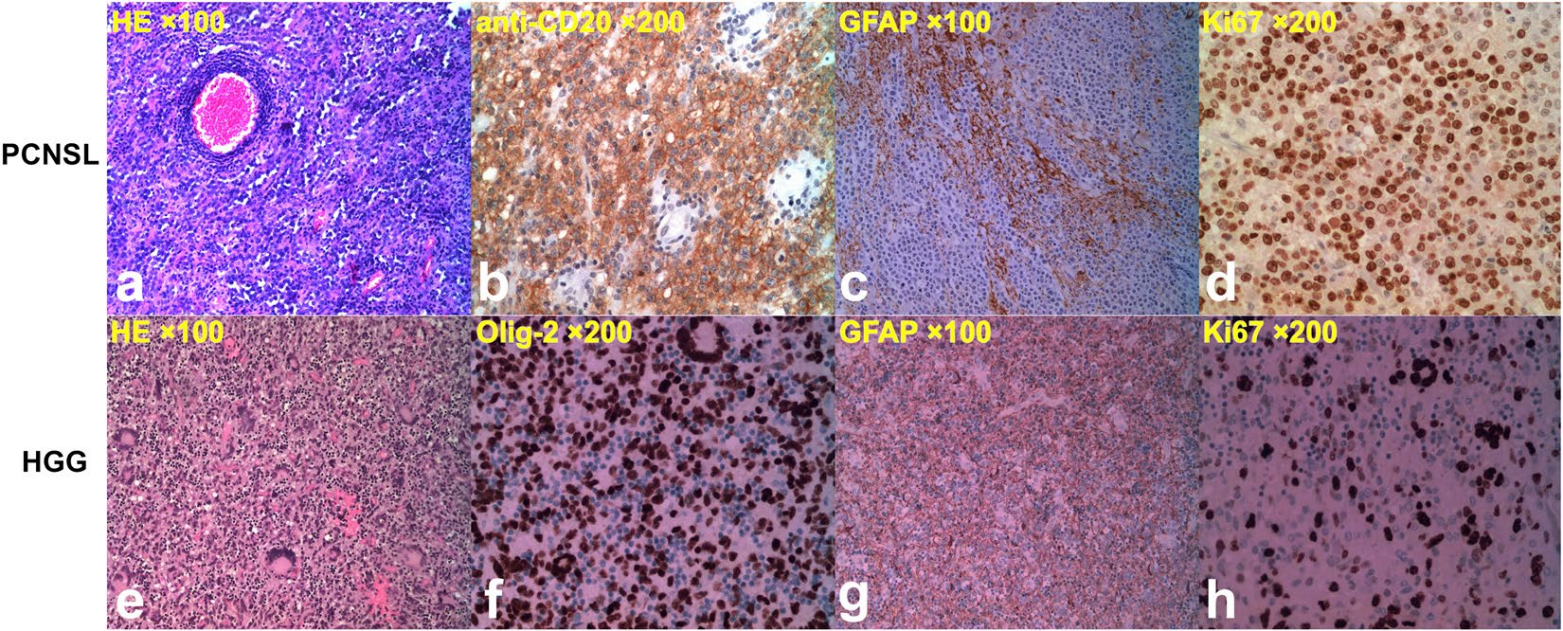
Pathological images of the same cases as in Fig. 4. **(a–d**) Diffuse large B cell lymphoma. **(e–h)** glioblastoma. **(a)** The central nervous system parenchyma was diffusely infiltrated by discohesive cells. The tumor cells were round and hyperchromatic, with scant cytoplasm and karyokinesis. The tumor cells were also demonstrated in the perivascular space like cuffs (H&E staining, ×100). **(b)** Immunohistochemistry, the tumor cells were positive for CD20 (anti-CD20, ×200). **(c)** Residual neuroglia was positive for GFAP, suggesting that the tumor cells grew along the white matter fiber (GFAP, ×100). **(d)** Immunohistochemistry, the Ki-67 index was higher than 90% (Ki-67 antibody, ×200). **(e)** The tumor cells were diffusely distributed, with different sizes, hyperchromatic nuclei, obvious heteromorphism and multinucleated giant cells (H&E staining, ×100). **(f)** Immunohistochemistry, the tumor cells were positive for oligodendrocyte transcription factor 2 (Olig-2, ×200). **(g)** Residual neuroglia was positive for GFAP (GFAP, ×100). **(h)** Immunohistochemistry, the Ki-67 index was about 50% (Ki-67 antibody, ×200).

At time of initial MRI, each lesion was evaluated for MR findings on all conventional sequences (Table 2). The representative cases of PCNSL and HGG were shown in Figs. 2–5.

Overall, tumor size, enhancement patterns, the presence of cystic changes, edema degree and streak-like edema were significantly different between PCNSL and HGG group (all P < 0.01). Both tumor maximum and mean diameter in PCNSL patients were smaller than ones in the HGGs (maximum diameter: 2.61 vs. 3.85 cm, P = 0.001; mean diameter: 2.22 vs. 3.22 cm, P = 0.002). Notably, homogenous enhancement was found in all 12 PNCSL patients, in contrast to heterogeneous enhancement detected in 100.0% of HGG lesions (P < 0.001). In addition, branch-like enhancement along white matter fiber was found in 8 (66.7%) cases of PNCSL, but none in HGG. Conversely, cystic change was present only in 2 (16.7%) case of PCNSL but in 14 (93.3%) HGG cases (P < 0.001). Regarding the degree of edema, the distribution of mild, moderate, and severe edema were 3 (25.0%), 6 (50.0%) and 3 (25.0%) cases in PCNSL, and 13 (86.7%), 1 (6.7%) and 1 (6.7%) cases in HGG, respectively. As for edema characteristics, streak-like edema in 7 cases of PNCSL was detected, but none in HGG patients (P < 0.01).

There were no significant differences in lesion type, the presence of bleeding, and involvement of brain surface between two groups (P = 0.554, 0.657 and 0.157, respectively). On lesion type, 2 (16.7%) and 5 (33.3%) of solitary infiltrative lesions, 2 (16.7%) and 0 of multiple demarcated lesions, 7 (58.3%) and 6 (40.0%) of solitary infiltrative lesions, 1 (8.3%) and 4 (26.7%) of diffuse infiltrative lesions were observed in PCNSL and HGG patients, respectively. Four (33.3%) PCNSL and 5 (33.3%) HGG patients appeared as tumor bleeding, and bleeding in all 4 cases of PCNSL was distributed in the periphery of the tumor. In case of brain surface involvement, PCNSL reached the brain surface (6 (50.0%) case) with meningeal infiltration present in 1 cases and ependymal infiltration in 1 cases, and HGG reached the brain surface (10 (66.7%) case) with meningeal infiltration present in 2 cases and ependymal infiltration in 2 cases.

### Inter-reader variability for ADC value

The inter-observer ICC value of ADC_min_ was close to 1 (0.947, P < 0.001), and the ICC values of ADC_total_ was higher than 0.75 (0.910, P < 0.001), which suggests very good measurement reliability of ADC values.

## Discussion

Cerebellar PCNSL or HGG is exceedingly rare diseases in adults, and accurate pretreatment differentiation is critically important to make therapeutic decisions. Current study analyzed the conventional MR and DW imaging features of PCNSL or HGG in adult cerebellum. As a result, we detected significant differences in ADC values, tumor size, enhancement patterns, the presence of cystic changes and streak edema between PCNSL and HGG patients. These findings highlight importance of combining conventional MR and DW imaging in diagnosing cerebellar PCNSL or HGG.

ADC measurements are useful in the differentiation between lymphoma and GBM. A main body of previous literature suggested that ADC values were significantly lower in PCNSL than in GBM patients owing to the increased cellularity^10–13,17^, and PCNSL has higher cellularity and nuclear-cytoplasm ratio than GBM^18^. Similarly, we also detected a significantly decreased ADC value in PCNSL than HGG in this study. ADC values are significantly dependent on ROI method. It has been suggested that ADC_min_ reflects an area with the highest tumor cellularity or the most proliferative portion of a tumor within heterogeneous tumors^14,19,20^. In this study, two methods of drawing ADC values were taken to obtain the relative minimum ADC (ADC_min_) value of the tumor and the average ADC (ADC_total_) value of the whole tumor. Considering the data reliability, the ICC analysis of ADC values from two observers was analyzed and good consistency between two readers was revealed. The results confirmed significant differences both in ADC_min_ and ADC_total_ values between PCNSL and GBM, which might aid in differentiating these two types of tumors. Although ADC could be useful to differentiate the PCNSL and HGG, in the clinical situation it is sometimes difficult to distinguish the two by ADC alone because of the overlap between them.

Conventional MR imaging is capable of differentiating between PCNSL and GBM lesions at time of initial presentation^2^. In current study, tumor size, enhancement patterns, the presence of cystic changes and streak edema were significantly different between two groups. The maximum and mean diameter in HGG patients were significantly larger than those in PNSSLs, which were not reported in previous research. Enhancement pattern is the most striking difference between PCNSL and HGG^2^. Consisted with previous reports^2,21^, we found homogeneous enhancement in all PCNSL and heterogeneous enhancement in all HGG patients, which attributed to the intra-tumor cystic change. Because all PCNSLs in this cohort were immunocompetent patients, no cystic changes occurred, post-contrast imaging showed homogeneous enhancement. On the contrary, marked cystic components revealed in 93.3% cases of HGG patients, it lead to a heterogeneous enhancement^22^. In addition, branch-like enhancement was found in 66.7% of PCNSLs, but none in HGG patients. Histologically, residual neuroglia was positive for GFAP in PCNSL, suggesting that the tumor cells grew along the white matter fiber, which may be the main reason for the branch-like enhancement along the white matter fiber in PCNSL. Therefore, the detection of branch-like enhancement may potentially be useful as a marker for PCNSLs.

Interestingly, in our study we detected that streak-like edema appeared in 58.3% of PCNSLs, but none in HGG patients. Histologically, tumor cells of PCNSL infiltrate along the white matter fiber, and this causes peritumoral edema also distributing along the white matter fibers. Based on anatomical histology, many parallel sulci on the surface of the cortex divides the cerebellum into many transverse folia and the white matter of the cerebellum is wrapped by the cortex. Therefore, the peritumoral edema along the white matter is also separated by the sulcus, appeared as streak-like sign on the axial MR images.

Although no significant differences were found in intratumoral bleeding, unlike the intratumoral appearance of HGG, it is confirmed that the characteristic peritumoral scattered bleeding in 4 cases of PCNSL, which may be related to the white matter infiltration of the tumor.

Our study has some limitations. Firstly, the sample size was relatively small, mainly because of the very low incidence of PCNSL or HGG in adult cerebellum, which may affect the effectiveness of statistics. Secondly, only one reader analyzed the conventional MR findings and calculated ADC value by drawing ROIs of focal tumor areas, which might cause sampling bias. Thirdly, due to its retrospective nature, we did not have sufficient data for reviewing MR spectroscopy or perfusion, which is valuable in differentiating these two types of tumor. Finally, limited to immunocompetent patients of this cohort, the MR imaging features of PCNSL in the immunocompromised are far more varied, and further research is warranted.

In conclusion, using the largest sample up to date, our results suggest that several conventional MR features (which are largely never reported before, including risk of aggressiveness,) such as enhancement patterns, branch-like enhancement and streak-like edema may be useful for the differentiation of PCNSL and HGG in cerebellum and, together with the quantitative measurements of ADC values, further improve the radiologists’ confidence for providing a better guidance for appropriate therapeutic strategies. Further studies are needed to support our results.
